# Influence of Indium (III) Chloride on Human Dermal Fibroblast Cell Adhesion on Tantalum/Silicon Oxide Nano-Composites

**DOI:** 10.3390/ma15103577

**Published:** 2022-05-17

**Authors:** Ali Eskandari, D. Moira Glerum, Ting Y. Tsui

**Affiliations:** 1 Department of Chemical Engineering, University of Waterloo, Waterloo, ON N2L 3G1, Canada; ali.eskandari@uwaterloo.ca; 2 Waterloo Institute for Nanotechnology, University of Waterloo, Waterloo, ON N2L 3G1, Canada; 3 Department of Biology, University of Waterloo, Waterloo, ON N2L 3G1, Canada

**Keywords:** human dermal fibroblast cell, adhesion, indium chloride, tantalum, biomaterial

## Abstract

Cell adhesion is an essential biological function for division, migration, signaling and tissue development. While it has been demonstrated that this cell function can be modified by using nanometer-scale surface topographic structures, it remains unknown how contaminants such as indium (III) ion might influence this specific cell behavior. Herein, the influence of indium chloride on human dermal fibroblast (GM5565) adhesion characteristics was investigated, given the frequent contact of contaminants with skin. The morphology of the adherent cells and their mitochondrial reticulum was characterized on cell culture dishes and nanopatterned surfaces by using fluorescence confocal microscopy and scanning electron microscopy. Results showed a significant proportion of cells lost their ability to align preferentially along the line axes of the nanopattern upon exposure to 3.2 mM indium chloride, with cells aligned within 10° of the pattern line axes reduced by as much as ~70%. Concurrent with the cell adhesion behaviors, the mitochondria in cells exposed to indium chloride exhibit a punctate staining that contrasts with the normal network of elongated tubular geometry seen in control cells. Our results demonstrate that exposure to indium chloride has detrimental effects on the behavior of human fibroblasts and adversely impacts their mitochondrial morphology. This shows the importance of evaluating the biological impacts of indium compounds.

## 1. Introduction

Indium is a soft metal [1] commonly used in dental alloys for dental restoration purposes [2,3] and in metal joining/soldering processes. Other indium compounds, such as indium-tin-oxide (ITO), are widely used in flat panel displays and optical devices [4] on the basis of its unique physical, optical and electrical properties. With the proliferation of indium-containing products, the impacts of indium compounds on biological entities and their potential role in disease have drawn significant interest recently [5,6,7,8,9,10]. At the cellular level, Ahmad et al. [11] showed indium oxide nanocubes induce mitochondrial membrane potential loss and the production of reactive oxygen species (ROS) in human lung epithelial cells, with resultant cytotoxicity and apoptosis. Tsai et al. [12] investigated indium chloride (InCl_3_)-induced cytotoxicity, apoptosis and genotoxicity on RAW264.7 mouse macrophage cells through the production of intracellular reactive oxygen species (ROS). Their results showed that InCl_3_ induced significant apoptosis and necrosis at concentrations greater than 1 μM and 50 μM, respectively. In addition, at InCl_3_ concentrations greater than 5 µM, a significant number of micronuclei were observed, indicating that InCl_3_ results in genotoxicity and DNA damage in these cells. The cytotoxic effects induced by InCl_3_ in human oral keratinocytes (IHOK), HSC-2 and SCC-15 were studied by Lee et al. [2], with their results showing a significant decrease in cell viability when cells were treated with InCl_3_ dosages greater than 1.6 mM; at 12.8 mM InCl_3_, cell viability decreased to less than 20%. Lee et al. also detected increased intracellular ROS formation with InCl_3_ dosages.

While prior studies [2,11,12] have shown some indium compounds can modify mitochondrial function and reduce cell viability, as well as increasing ROS production in a variety of human and mammalian cell types, there is little understanding of how indium might affect the adhesive behavior and the morphology of mitochondria in human fibroblasts cultured on engineered surfaces. Engineered surfaces are used to induce desired cell alignment on medical implants [13,14,15] and scaffolds [16] in order to promote cell adhesion [17,18,19], inflection control [15,20], regeneration [21], differentiation [19,22] and osteointegration of implants [21,23]. Custom-designed topographic features have also been created on biomedical devices for tissue engineering [16], migration control [17] and cell immobilization [12]. While the majority of these prior works used fabricated engineered surface structures with monolithic material, advanced 3D nano-composite surface structures consisting of dissimilar materials [24] (such as tantalum and silicon oxide) have been successfully fabricated only recently by using advanced integrated circuit manufacturing techniques, and they have been found to exhibit excellent cell alignment behaviours. These composites use both surface topographic cues and cell adhesive behavior to manipulate both cell morphology and orientation. Moussa et al. [24] showed mammalian cells preferred to adhere to tantalum rather than silicon oxide. However, it remains unclear if cellular behaviours on these surfaces are sensitive to contaminants or additives in the medium, such as indium compounds. Moussa et al. [25] have shown that a small amount (5 μM) of the bacterial toxin Antimycin A in the medium is sufficient to reduce the human fibroblast cell alignment on engineered tungsten surfaces by as much as 35% and degrade the functionality of the device. Therefore, it is important to understand the impact of potential contaminants or additives in the media on the cell alignment effectiveness on these new nano-composite surface structures.

Mitochondrial morphology is an important indicator of cellular physiology [26,27], and disruptions of morphology have been documented under conditions of oxidative stress [27], with mitochondrial fragmentation being related to hyperglycemia-induced ROS production. In addition to changes in mitochondrial morphology, prior studies [28,29] have shown ROS play an important role in the focal adhesion pathway, acting as a mediator for integrin signaling and spreading in fibroblasts. Given the demonstrated role of InCl_3_ in inducing ROS production, we hypothesized that the introduction of InCl_3_ in cell culture media for dermal fibroblasts could lead to alterations in the normal adhesive behavior of the cells and induce discernible changes to the mitochondrial morphology.

The objective of this work was to investigate how human fibroblasts react on nanopatterned surfaces consisting of tantalum/silicon oxide parallel line/trench features, upon exposure to InCl_3_. The line/trench structures have widths in the range of 0.18 µm to 10 µm, and tantalum is a bioactive metal often used in orthopedic implants [14,30]. Because the cell adhesion and alignment mechanisms on these nano-composite surfaces is considerably more complex than on flat surfaces composed of monolithic material, such as petri dishes, detailed characterization of cell behaviors on these engineered surfaces is required. Cells were cultured either without indium or with 3.2 mM InCl_3_ added to the media. Indium (III) chloride has been used as an indium ion precursor in prior toxicity studies [2,8,9], and we hypothesized that the excess ROS production in cells induced by the InCl_3_ [2] would result in changes to cell morphology, alignment behavior and mitochondrial morphology on the engineered surfaces. In this work, cell alignment relative to the patterned line axes and mitochondrial morphology were quantified using fluorescence confocal microscopy and high-resolution scanning electron microscopy (SEM). We observed unique changes in cell and mitochondrial morphology after exposing cells to 3.2 mM InCl_3_. Overall cell shape became more rounded, while the morphology of the mitochondria shifted from a tubular distribution to a more punctate staining pattern.

## 2. Materials and Methods

### 2.1. Substrates

**Figure 1:**
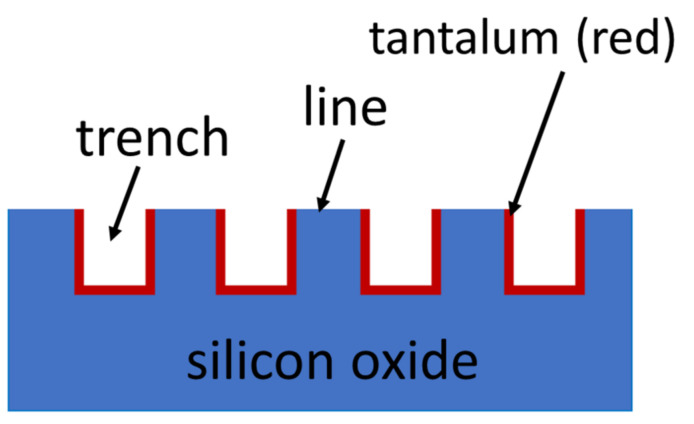
Schematic cross-section drawing of the parallel line/trench structures tested in this work. The trench sidewall and bottom surfaces are covered with a tantalum thin film where the silicon oxide is exposed on the top surface. Line and trench widths are equal.

Behavior of adherent cells was characterized on two different substrates in this work —(i) 35 mm tissue culture dishes (Biolite 35 mm, Thermo Scientific, Mississauga, ON, Canada) and (ii) tantalum/silicon oxide nanocomposites with parallel line comb surface structures. A schematic cross-section drawing of the comb structure is illustrated in Figure 1. It shows the structure consists of lines and trenches of equal widths. The trench depth of all samples is identical at ~0.5 µm. The sidewall and bottom surfaces of the trenches are covered with tantalum thin film, while the top surface of lines consists of silicon oxide. Specimens with six different line/trench widths of 0.18 µm, 0.25 µm, 0.5 µm, 1 µm, 2 µm and 10 µm were prepared. Detailed fabrication processes for the nanocomposites have been described elsewhere [24].

### 2.2. Cell Culture and Plating

Human skin fibroblasts (commercial cell line GM5565; from ATCC/Coriell Institute, Camden, NJ, USA) were cultured in a medium consisting of Minimum Essential Medium Alpha (Gibco/Thermo Fisher Scientific) and supplemented with 10% (*v*/*v*) fetal bovine serum (FBS). Cells were maintained in T-50 flasks with 10 mL of media, grown at 37 °C and 5% CO_2_. The media contain less than 0.5 µM of indium ions, as characterized by inductively coupled plasma mass spectrometry. Two different media solutions were prepared: indium-free media, containing only the chemical components described above and media supplemented with 3.2 mM of indium (III) chloride (99.99%, Sigma-Aldrich, Oakville, ON, Canada). Prior to cell seeding, nanocomposite substrates were sterilized with 2 mL of 70% ethanol for 1 min and then air dried and rinsed with 2 mL of sterile phosphate-buffered saline (PBS) twice to eliminate any ethanol residue. Substrates were inoculated with media containing 2–9 × 10^4^ cells/mL and incubated in 6-well tissue culture plates (VWR^®^ Multiwell Cell Culture Plates, 10062-892) for 24–48 h at 37 °C and 5% CO_2_.

### 2.3. Fixation and Staining

After 24–48 h of incubation, media was removed, and the adherent cells were rinsed with PBS. Mitochondria in live cells were stained by exposure to 2 mL of 100 nM pre-warmed Mitotracker^TM^ Red (Thermo Fisher Scientific, Mississauga, ON, Canada) at 37 °C for 20 min. After rinsing the specimens with 2 mL of PBS, cells were fixed with 4% paraformaldehyde for 15 min. To prepare cells for additional staining, they were permeabilized with 2 mL of 0.1% Triton-X (Sigma-Aldrich, St. Louis, MO, USA) for 5 min, followed with PBS rinses. F-actin microfilaments were labeled with Phalloidin-iFluor 647 Reagent (ab176759, Abcam Inc, Cambridge, MA, USA) by exposing cells to the reagent for 45 min, followed with 3 rinses with PBS. DNA was labeled with 4′,6-diamidino-2-phenylin-dole (DAPI) (Life Technologies, Burlington, ON, Canada) by submerging cells in a 1:25,000 DAPI dilution in PBS for 5 min. The final step of sample preparation includes rinsing the stained cells with PBS multiple times and storage of the samples at 4 °C. Fluorescence confocal microscopy was carried out at the University of Guelph (Guelph, ON, Canada) using a Leica TCS SP5 confocal microscope (Leica, Wetzlar, Germany).

### 2.4. Scanning Electron Microscopy

After the paraformaldehyde fixation process described above, specimens were dehydrated by submerging in increasing concentrations of ethanol (50%, 75%, 95%, and 100%) for at least 10 min each. Dehydrated specimens were inspected using a high-resolution field-emission scanning electron microscope (Zeiss 1550, Carl Zeiss AG, Oberkochen, Germany) with electron gun voltage operating at 7 kV.

### 2.5. Cell Orientation Measurements

The orientation of adherent cells on the engineered surfaces consisting of parallel line patterns was quantified by measuring the angle (φ) between the long axis of elliptical-shaped nuclei and the pattern axes. A schematic drawing of this angular displacement is illustrated in Figure S1. These cell orientation measurements were conducted on the SEM micrographs. The orientation of 100 randomly selected cells from each pattern geometry were characterized manually by using the Angle Tool of the ImageJ software (U.S. National Institutes of Health, Bethesda, MD, USA).

## 3. Results and Discussions

### 3.1. Cell and Mitochondrial Morphology on Flat Surfaces

**Figure 2:**
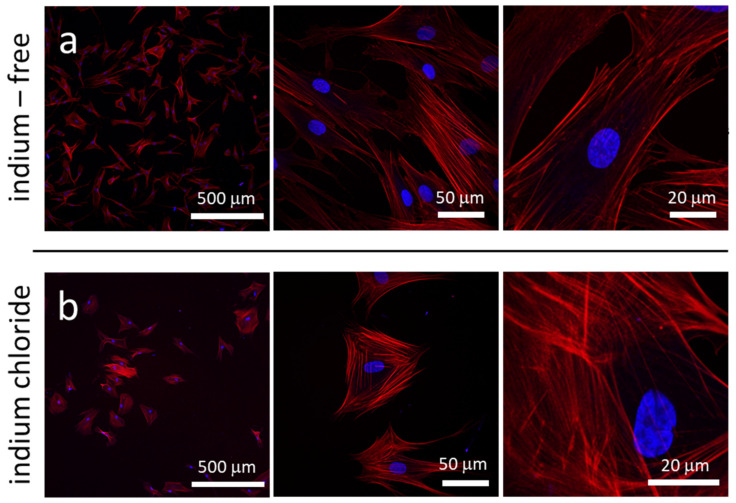
Fluorescence confocal micrographs of adherent cells in petri dish prepared with (**a**) indium-free media and (**b**) media containing 3.2 mM of InCl_3_. Images show cell density decreases after they are exposed to indium chloride. Cells also become more compact and circular in shape. DNA molecules are visualized with DAPI in blue; actin filaments are stained with deep red Cytopainter F-Actin.

We first sought to characterize the behavior of the cells and their mitochondria on the regular substratum provided by the plastic culture dishes. Representative fluorescence confocal micrographs of human dermal fibroblasts (GM 5565) incubated on flat culture dish surfaces in medium either without indium or containing 3.2 mM of indium (III) chloride (InCl_3_) for 24 h are displayed in Figure 2a,b, respectively. Specimens were imaged at three different magnifications, with DNA labeled by blue DAPI and the actin microfilaments by red CytoPainter. Figure 2 reveals the number of adherent cells per unit area (cell coverage density) decreases significantly when cells were exposed to InCl_3_. Micrographs show cells prepared in an indium-free environment (Figure 2a) have elongated dendrite structures and are oriented randomly. In contrast, cells treated with InCl_3_ are more compact, exhibit fewer dendritic projections and are more circular in shape (Figure 2b). To quantify the degree of roundness of these cells, the circularity index of each cell was calculated based on the following equation:

(1) $$circularity\;index\;\left(C\right)=4\pi \frac{cell\;area}{{\left(cell\;primeter\;length\right)}^{2}}$$

The circularity index is an unitless parameter with a value in the range from zero to one, with a value of one for a cell that is perfectly circular in shape. The circularity indices and surface coverage densities of adherent cells on the flat culture dish surface in the two different medium compositions are summarized in Table 1 (data spread corresponds to one standard deviation). Each circularity index value was calculated from the morphology of 50 randomly selected cells. Results show adherent cells treated with InCl_3_ have an average circularity value of 0.42, which is 55% larger than cells prepared in an indium-free medium with a circularity index of 0.28, indicating a rounding of cells upon indium chloride treatment.

In addition to cell morphology, the coverage density of cells on the flat culture dish surfaces was characterized and is shown in Table 1. The coverage density is the average value calculated from eight randomly selected square-shaped regions, with a sampling area of 2.4 mm^2^ each. The total number of cells (*n*) characterized in all eight regions is also reported in the table. Results show that coverage of cells on the petri dish surface decreased from 14.2 cells/mm^2^ to 9.2 cells/mm^2^ (~35% reduction) when treated with InCl_3_, which is consistent with the 40% decrease in viability of InCl_3_-treated human oral keratinocytes (IHOK) observed by Lee et al. [2].

**Figure 3:**
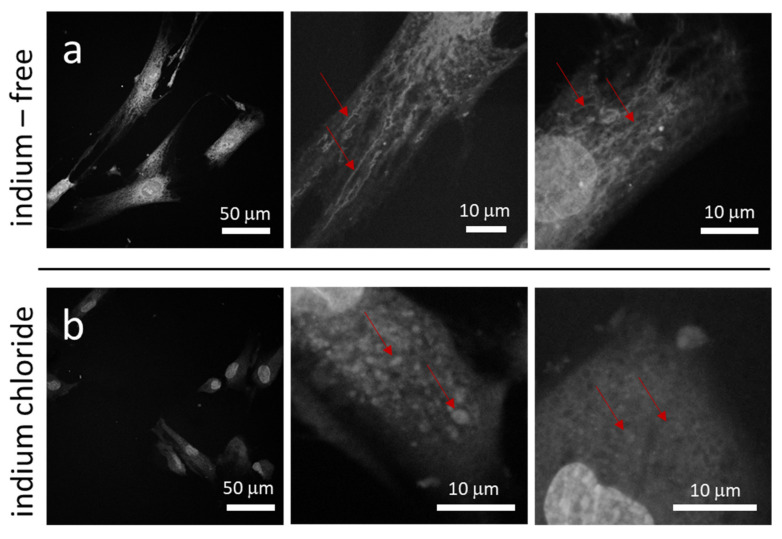
Fluorescence confocal micrographs of mitochondria within adherent cells in culture dish prepared in (**a**) medium without InCl_3_ and (**b**) medium containing 3.2 mM of InCl_3_. Images show long tubular mitochondria form networks in cells incubated in indium-free media. When treated with indium chloride, mitochondria are punctate in shape. The red arrows highlight stained mitochondria.

We next sought to determine whether indium chloride would have any effect on the mitochondrial morphology of the fibroblasts. Using MitoTracker Red to visualize the mitochondrial reticulum, the morphology of mitochondria in cells treated with indium-free and InCl_3_-containing media is displayed in Figure 3a,b, respectively. Figure 3a shows mitochondria form extended tubular networks in cells prepared with indium-free media. In contrast, mitochondria in cells exposed to InCl_3_ are fragmented and acquire a punctate geometry that suggests the organelles may be aggregating (see Figure 3b). The mitochondrial morphological change from a tubular network to puncta was also observed by Moussa et al. [25] when GM5565 cells were exposed to Antimycin A, an inhibitor of Complex III of the mitochondrial respiratory chain that leads to an increase in ROS production. This result thus suggests that the increased production of ROS induced by indium chloride may play an important role in the altered mitochondrial morphology.

### 3.2. Cell and Mitochondrial Morphology on Engineered Surfaces

**Figure 4:**
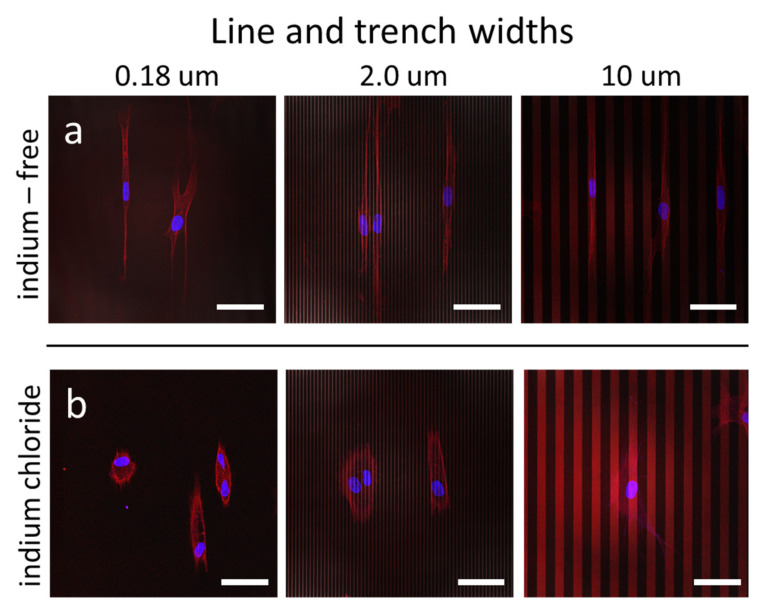
Fluorescence confocal micrographs of adherent cells on parallel line/trench structures incubated in (**a**) medium without InCl_3_ and (**b**) medium containing 3.2 mM of InCl_3_. Images show cells elongated and aligned to the line axes when cultured in baseline media without indium chloride. After exposure to the indium chloride, cells are more compacted and less likely to align to the pattern axes. Scale bars correspond to 50 µm. Cell nuclei appear blue (4′,6-diamidino-2-phenylindole; DAPI), whereas F-actin microfilaments appear red (fluorescent phalloidin conjugate).

The interplay between indium chloride, engineered surface structure geometry and adherent cell morphology was then studied, as revealed in the fluorescence confocal micrographs displayed in Figure 4. DNA molecules are labelled in blue (DAPI), while actin micro-filaments are visualized in red. The micrographs show the adherent cells on parallel line structures with equal line and trench widths of 0.18 µm, 2 µm and 10 µm; as shown in Figure 4a, adherent cells prepared with indium-free media were elongated and oriented parallel to the line axes on all three topographic features. Adherent cells on the patterns with widths of 0.18 µm and 2.0 µm covered multiple lines and trenches, whereas cells elongated and fit into a single trench on the structures with 10 µm line width. In sharp contrast, the adherent cells treated with indium chloride (Figure 4b) were compact and acquired an elongated oval shape. Detailed inspections of these micrographs reveal thin-short filopodia protruding from these cells, with some of these extended along the line direction, demonstrating that the adherent cell geometry on engineered surfaces is also affected by indium chloride exposure. High magnification and resolution micrographs of F-actin microfilaments on the pattern with widths of 0.18 µm and 2.0 µm are displayed in Figure S2. They show these microfilaments extend along the line axes, regardless of the indium chloride concentration in the media. For cells adhered on the 2.0 µm structure, images show microfilaments preferentially elongated in the trenches. Additional fluorescence confocal micrographs of adherent cells on structures with line widths of 0.18 µm, 0.25 µm, 0.5 µm, 1 µm, 2 µm and 10 µm are displayed in the supplementary figures, both for cells incubated in medium without indium chloride (Figure S3) and in the presence of 3.2 mM indium chloride (Figure S4). These micrographs show that the majority of cells were elongated and aligned to the line axes when cells were incubated in the indium-free medium (see Figure S3). However, cells are more rounded when exposed to 3.2 mM of indium chloride, as shown in Figure S4. These cell behaviors are consistent with those observed in Figure 4 and on the flat culture dish surfaces.

**Figure 5:**
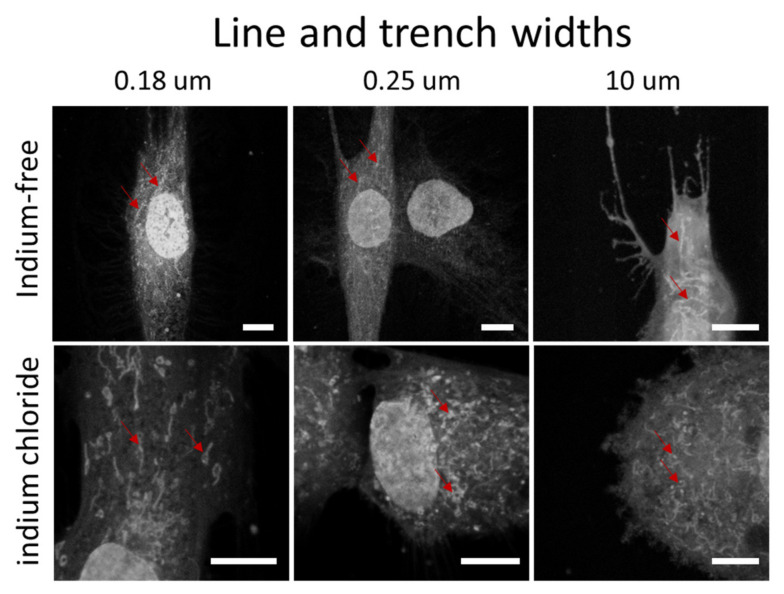
Fluorescence confocal micrographs of adherent cells stained with Mitotracker^TM^ on parallel line/trench structures with widths of 0.18 µm, 0.25 µm and 10 µm. Cells were cultivated in two different medium compositions—without and with indium chloride. Scale bars correspond to 10 µm. The red arrows highlight some of the mitochondria.

The morphology of mitochondria within cells on the engineered surfaces was also characterized. Representative fluorescence confocal micrograph of adherent cells on structures with line/trench widths of 0.18 µm, 0.25 µm and 10 µm are shown in Figure 5 and show that mitochondria assume a tubular shape and form networks within cells cultured in medium without indium chloride. In contrast, mitochondria are more fragmented and exhibit a punctate geometry when exposed to the metal. This is consistent with the observation by Moussa et al. [24] that mitochondrial morphology changed from tubular to punctate when cells were treated with the ROS-inducing agent Antimycin A. Our present results suggest that the known role for indium in increasing cellular oxidative stress has a similar impact on mitochondrial morphology.

### 3.3. Cell Alignment Characteristics on Engineered Surfaces

**Figure 6:**
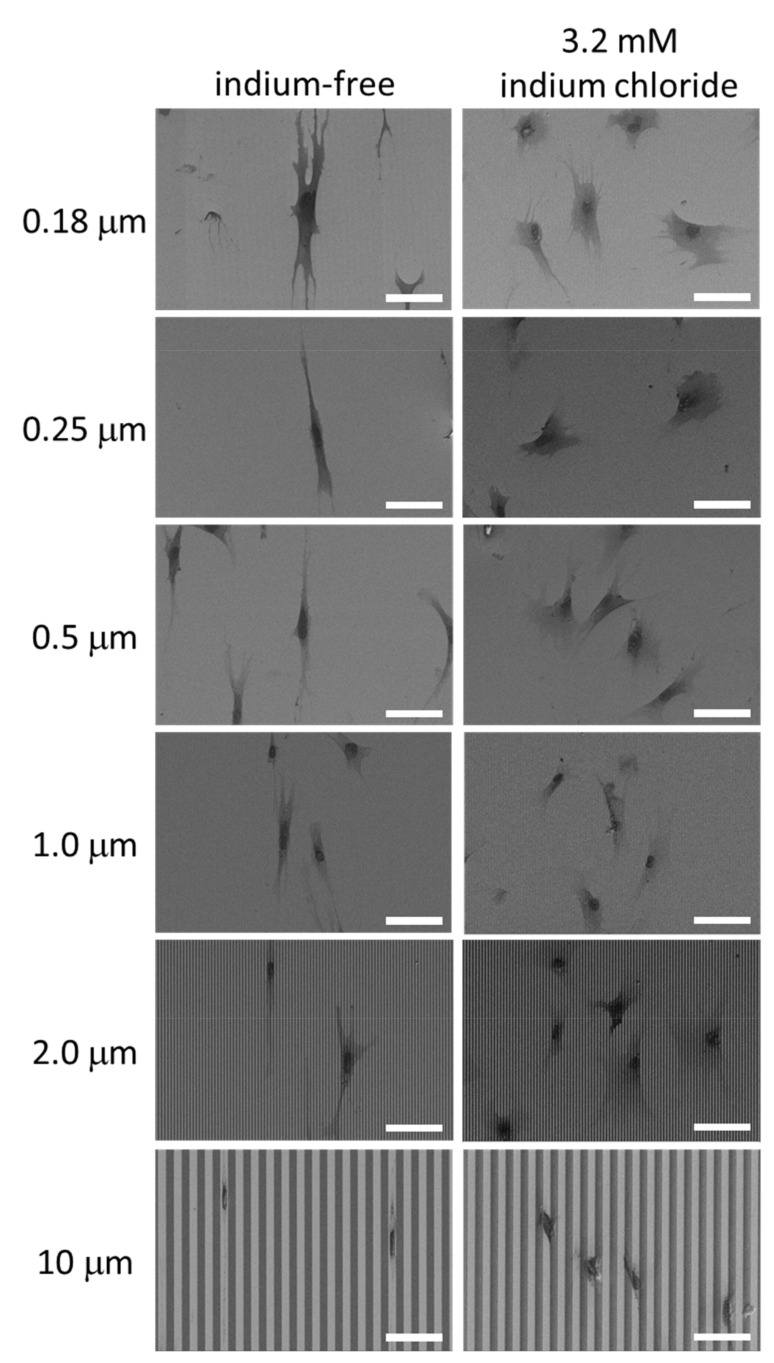
Typical SEM micrographs of adherent cells on patterned structures after incubated in indium-free media and media containing chloride media. Scale bars correspond to 75 μm.

In addition to the cell morphology, the cell alignment behavior on the parallel line surface structures was also studied. Typical scanning electron micrographs of adherent cells on 0.18 µm, 0.25 µm, 0.5 µm, 1 µm, 2 µm and 10 µm parallel line structures, incubated in indium-free and indium chloride-containing media, are displayed in Figure 6. The lines and trenches are aligned vertically in these images. Results show that the majority of adherent cells incubated in the absence of indium chloride were aligned to the line axes, whereas cells treated with indium chloride oriented in arbitrary directions and were more rounded.

**Figure 7:**
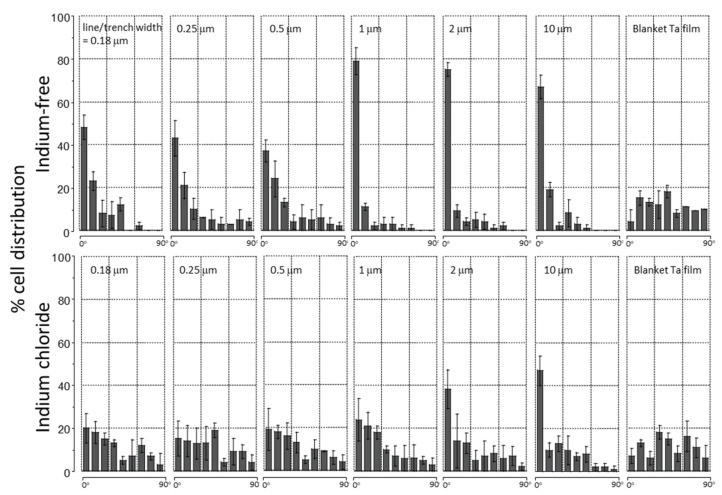
Percent distribution of cell aligned on parallel line structures with equal line/trench widths of 0.18 µm, 0.25 µm, 0.5 µm, 1 µm, 2 µm and 10 µm. The orientation of 100 randomly selected cells was measured in each geometric patterned area. The data spread corresponds to one standard deviation. The top and bottom panels display results from cells cultured in medium without and with indium chloride, respectively.

As we have described in prior work [31,32,33], cell alignment behavior on parallel line/trench topographic structures can be quantified by measuring the angle between the long axis of the nucleus and the pattern line axes. Figure 7 shows the percent cell distribution results of six different patterns with equal line and trench widths of 0.18 µm, 0.25 µm, 0.5 µm, 1 µm, 2 µm and 10 µm, both in cells cultured in the presence (bottom) and absence (top) of indium chloride, respectively. As a comparison, alignment results of adherent cells on blanket tantalum films are also included in this figure. The orientation of 100 randomly selected cells was measured in each patterned structure. The data spread represents one standard deviation calculated from three different independent measurements. Each bin corresponds to a 10° increment of angular displacement—e.g., the second bin represents the population of cell with the nuclear long axis aligned from 10° to 20° and −10° to −20° of the line axes. Our results show the population of cells aligned within the ±10° angular displacement are pattern dependent. For cells cultured in indium-free media, ~37% to ~48% of the cell population is aligned to within 10° of the line axes on line/trench structures with widths of 0.18 µm, 0.25 µm and 0.5 µm, while structures with a line/trench width of 1 µm induce the strongest cell alignment, with ~79% of the cell population oriented within ten degrees of the line axes. Interestingly, a decreased percentage of the cell population, ~75% and ~67%, is aligned to the line axes on structures with the larger line/trench widths of 2 µm and 10 µm, respectively.

**Figure 8:**
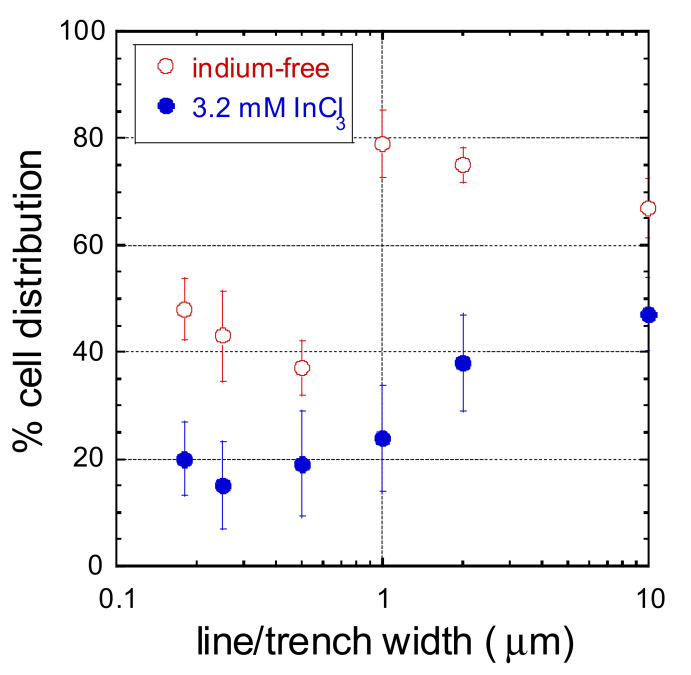
Percent cell distribution of cells aligned within ± 10° of line axes as a function of line width.

The influence of indium chloride on cell alignment performance on structures with specific line/trench width is highlighted by comparing the differences in alignment in the absence (upper panel) and presence (lower panel) of the metal salt (Figure 7). When cells are treated with InCl_3_, there is a significant decrease in the percentage of cells that align to the line axes on structures with line/trench widths in the range of 0.18 µm to 1 µm. None of these narrow line structures is characterized by having more than 25% of the cells aligned to the line axes. For structures with line/trench widths larger than 1 μm, the decrease in cell population aligned to the line axes is line/trench width dependent. To highlight this pattern dependence, the average percent population of cells aligned within ±10º from the line axes is plotted as a function of line/trench width in Figure 8. The results show that the decrease in cell population aligned to the line axes on the structure with line width of 1 μm is ~2.8 times larger than those observed on the structure with a line width of 10 μm. This is believed to be the first demonstration in the literature of how the influence of indium chloride on cell adhesion behavior can be affected by the surface topography of the substratum.

One possible explanation for the reduced influence of indium chloride on cell alignment on the 10 µm wide line structure may be due to the difference between the cell-to-surface contact configurations on the narrow and wide-line/trench structures, likely influenced by the size of the cells themselves. Moussa et al. [20] observed that the majority of filopodia and lamellipodia of Vero (monkey kidney epithelial) cells only contact the top surfaces of lines and do not descend into the trenches with widths smaller than 2 µm. Instead, cellular components appeared to bridge the trenches and adhere to the surfaces, suggesting that the aberrant behaviour we have observed here may be due to a ROS-induced reduction in focal adhesion activities caused by the indium chloride. On structures with line/trench widths of 10 µm, Moussa et al. observed that the majority of the cell body descended into the trenches and conformed to the trench surface. While cell focal adhesion may be disrupted by indium chloride in these structures, the mechanical constraint produced by the trench may facilitate the fit of the cells into the line axes.

The new findings presented in this work suggest that it would be prudent to evaluate cell adhesion and alignment behaviors on medical implants or devices that contain indium alloys/compounds. The ability of the engineered surfaces to control the cell alignment may decrease or be compromised by the indium content. Furthermore, our results show that indium-induced alignment degradation is pattern dependent. The data also show, however, that it is still possible to control cell orientation or morphology in an indium chloride containing environment by using structures with line/trench width in the order of 10 μm, in which the cells appear to be less affected by the presence of indium.

## 4. Conclusions

Fluorescence confocal microscopy has demonstrated that adherent dermal fibroblasts growing on flat culture dish surfaces change their morphology, becoming more compact and circular in shape after exposure to indium chloride. The mitochondrial morphology in these cells also changed, from a tubular network to broadly dispersed puncta. The GM5565 cells were able to adhere and align to the tantalum/silicon oxide parallel line structures, with the distribution of cells that align to the line axes depending on the pattern geometry and as much as ~79% of the cell population oriented to within 10° of the line axes on structures with a line/trench width of 1 µm. The introduction of InCl_3_ to the medium significantly reduces cells’ abilities to align to the trenches and is most evident on the patterned substrates with line widths of 1 µm. Taken together, our results demonstrate quantifiable changes to both cellular and mitochondrial morphology in dermal fibroblasts exposed to indium chloride and support further investigation into the potential effects of indium contaminants on human health.

## Figures and Tables

**Table 1:** Adherent cell coverage density and circularity index of cells incubated in culture medium that is indium-free or contains 3.2 mM indium chloride. Data spread corresponds to one standard deviation.

Sample ID	Indium Chloride Concentration (mM)	Circularity Index	Surface Coverage Density (Cell/mm^2^)
1	0	0.28 ± 0.13	14.2 ± 2.9 (*n* = 273)
2	3.2	0.42 ± 0.18	9.2 ± 1.8 (*n* = 176)

## Data Availability

The data presented in this study are available on request from the corresponding author.

## Supplementary Materials

The following supporting information can be downloaded at: https://www.mdpi.com/article/10.3390/ma15103577/s1, Figure S1: Schematic drawing of an adherent cells on tantalum/silicon oxide parallel line structure. The angular displacement between the long axis of the elliptical-shaped nucleus and the line axis is defined as (φ). Figure S2: High-resolution fluorescence confocal micrographs of cells on structures with line/trench widths of 2 μm and 0.18 μm. The left four panels show cells incubated in indium-free media. The right four panels present cells treated with 3.2 mM indium chloride. Scale bars correspond to 20 μm. The lines and trenches are aligned vertically in these images. Figure S3: Fluorescence confocal micrograph of adherent cells on parallel line/trench structure with line widths of 0.18 µm, 0.25 µm, 0.5 µm, 1 µm, 2 µm and 10 µm. These cells were treated with indium-free medium. DNA molecules were stained with blue DAPI, while actin micro-filaments were labeled with red phalloidin. The lines and trenches are aligned vertically in these images. Scale bars correspond to 300 µm. The dash lines indicate the patterned areas. Figure S4: Fluorescence confocal micrograph of adherent cells on parallel line/trench structure with line widths of 0.18 µm, 0.25 µm, 0.5 µm, 1 µm, 2 µm and 10 µm. These cells were cultivated in media containing 3.2 mM of indium chloride. DNA molecules were stained with blue DAPI, while actin micro-filaments were labeled with red phalloidin. The lines and trenches are aligned vertically in these images. Scale bars correspond to 300 µm. The dash lines indicate the patterned areas.
