# Supplementary material for: Influence of Indium (III) Chloride on Human Dermal Fibroblast Cell Adhesion on Tantalum/Silicon Oxide Nano-Composites
Source: Materials (Basel). 2022 May 17;15(10):3577. doi: 10.3390/ma15103577 (PMC9147986; doi:10.3390/ma15103577)
Supplement: Supplementary file 1 [file materials-15-03577-s001.zip › materials-1689500-supplementary.pdf]

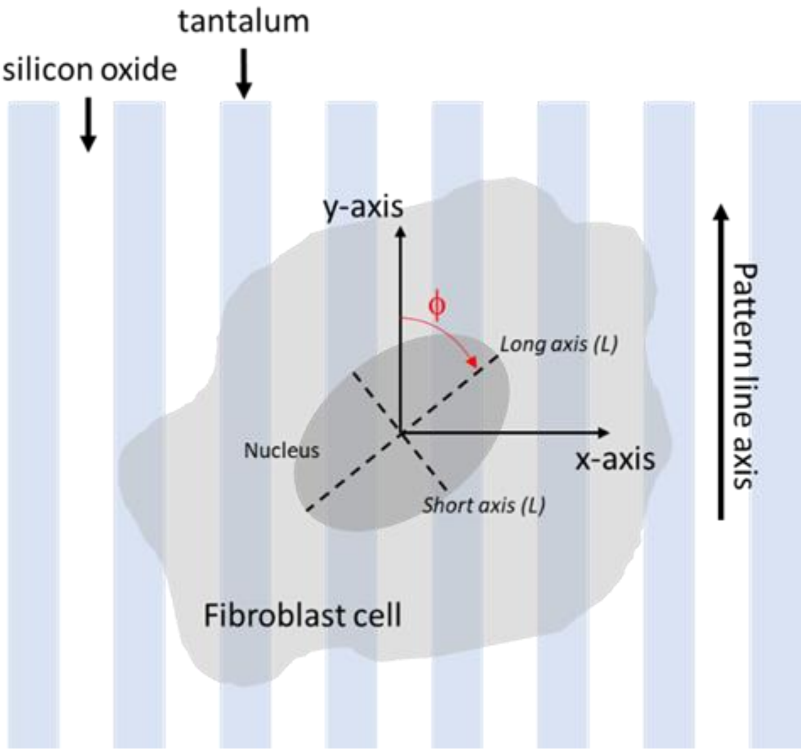

Figure S1. Schematic drawing of an adherent cells on tantalum/silicon oxide parallel line structure. The angular displacement between the long axis of the elliptical-shaped nucleus and the line axis is defined as (ϕ).

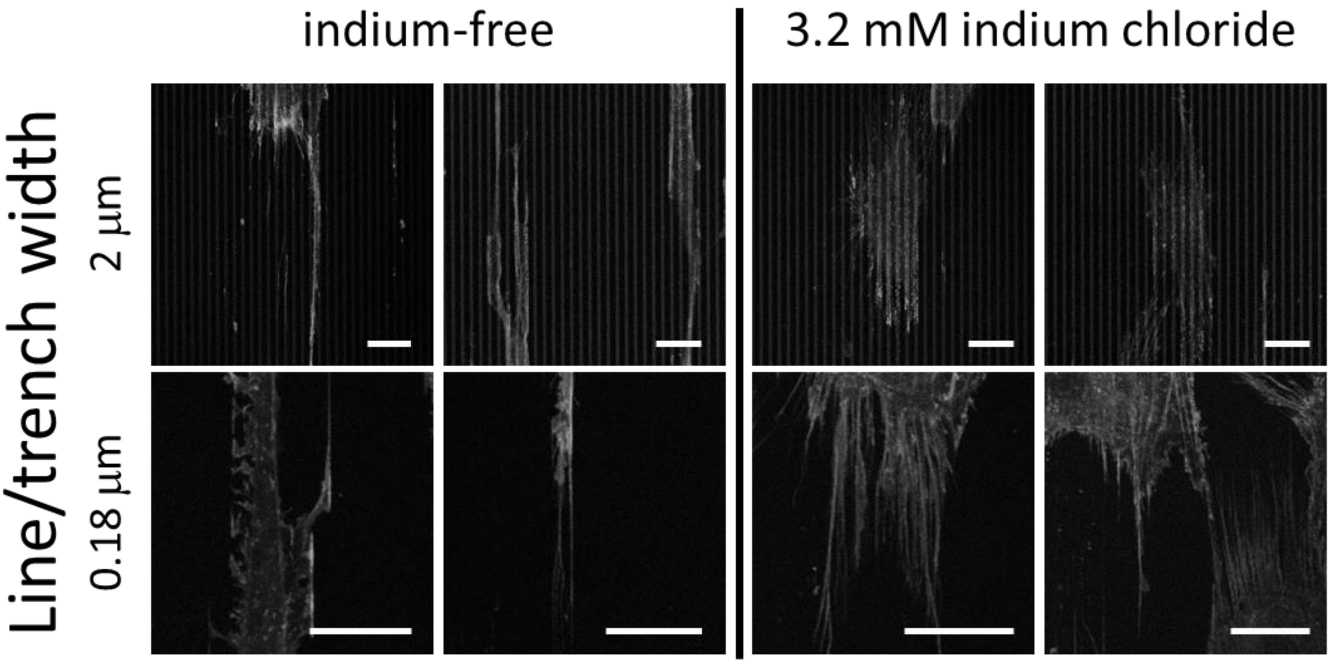

Figure S2. High-resolution florescence confocal micrographs of cells on structures with line/trench of 2 μm and 0.18 μm. The left four panels show cells incubated in indium-free media. The right four panels reveal cells treated with

3.2 mM indium chloride. Scale bars correspond to 20 μm. The lines and trenches are aligned vertically in these images.

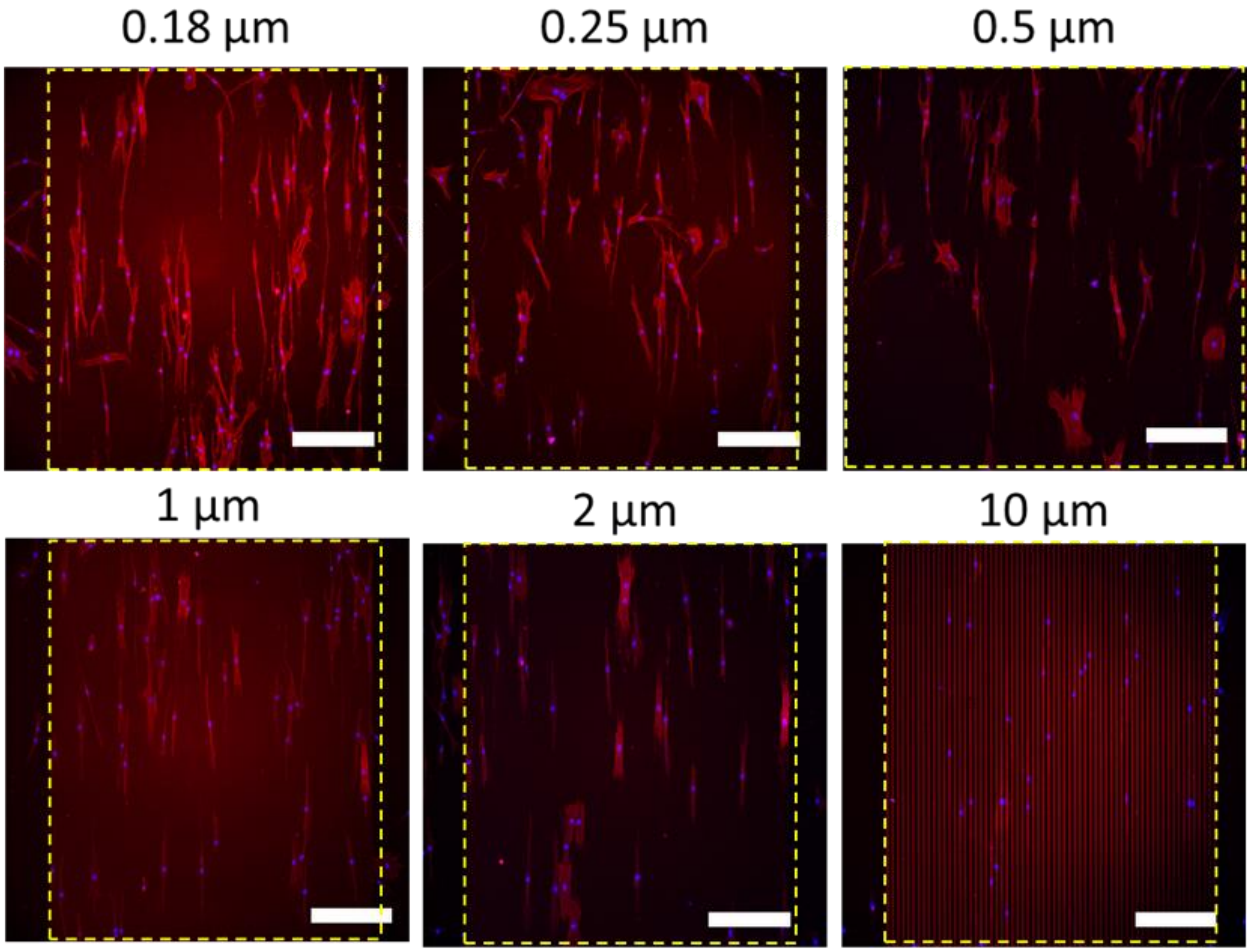

Figure S3. Fluorescence confocal micrograph of adherent cells on parallel line/trench structure with line widths of 0.18 μm, 0.25 μm, 0.5 μm, 1 μm, 2 μm, and 10 μm. These cells were treated with indium-free medium. DNA molecules were stained with blue DAPI while actin micro-filaments were labeled with red phalloidin. The lines and trenches are aligned vertically in these images. Scale bars correspond to 300 μm. The dash lines indicate the patterned areas.

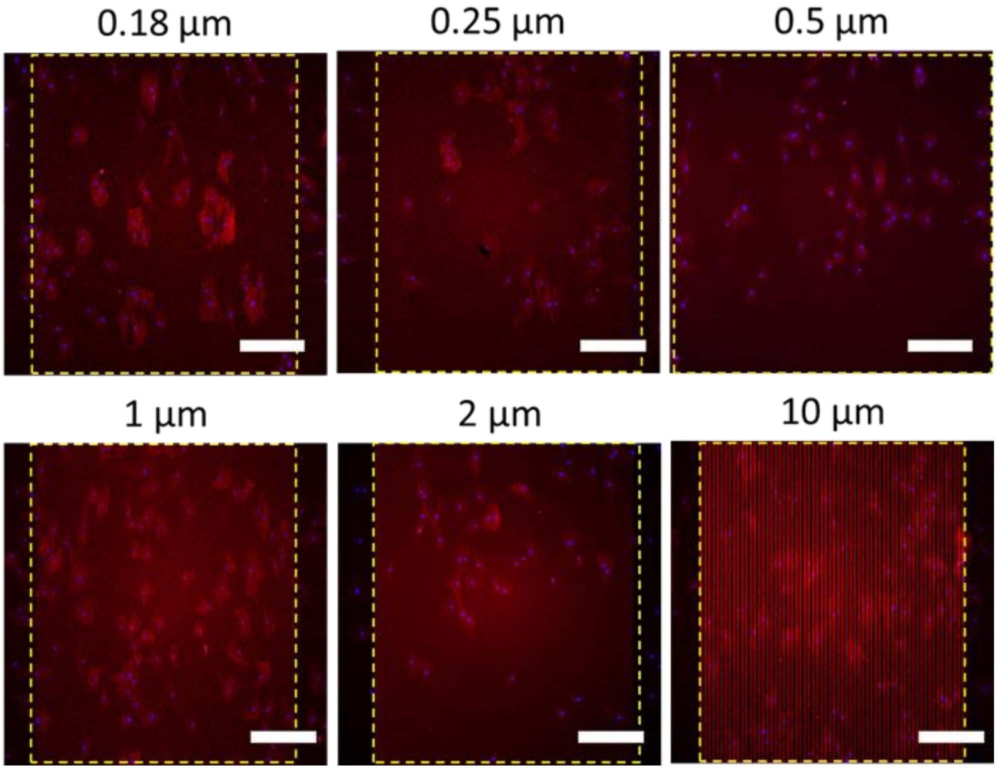

Figure S4. Fluorescence confocal micrograph of adherent cells on parallel line/trench structure with line widths of 0.18 μm, 0.25 μm, 0.5 μm, 1 μm, 2 μm, and 10 μm. These cells were cultivated in media containing 3.2 mM of indium chloride. DNA molecules were stained with blue DAPI while actin micro-filaments were labeled with red phalloidin. The lines and trenches are aligned vertically in these images. Scale bars correspond to 300 μm. The dash lines indicate the patterned areas.
